# Young people are not blameworthy: the generation’s awareness of COVID-19 and behavioral responses

**DOI:** 10.1038/s41598-021-03036-x

**Published:** 2021-12-08

**Authors:** Seung-Pyo Jun, Hyoung Sun Yoo, Chul Lee

**Affiliations:** 1 Korea Institute of Science and Technology Information and Science and Technology Management and Policy, University of Science and Technology (UST), Seoul, South Korea; 2 Korea Institute of Science and Technology Information, Seoul, South Korea

**Keywords:** Public health, Health policy, Disease prevention

## Abstract

At a time when the COVID-19 pandemic has been ongoing for more than a year, young people have been the subject of vigilant scrutiny and criticism regarding their active engagement in social activities. We posed the question of whether young people's response to COVID-19 was different from that of other generations and analyzed awareness and behavior to investigate this question. Specifically, we examined internet searches for information on COVID-19 and credit card consumption in South Korea among young people in their 20s and compared them to a reference group of people in their 50s. Our research has confirmed that there was no statistically significant difference between young people and the reference group in this regard. Furthermore, in the 25 sub-sectors of industry we examined, young people's consumption activities recovered significantly faster than the reference group in only three sub-sectors. This study demonstrated that young people showed stronger interest than the reference group in their response to COVID-19, and that they cooperated with the government’s social distancing policy by reducing their activities. Through this study, we presented a scientific approach for evaluating young people in regard to their response to COVID-19, offering useful implications for designing appropriate policies for public health.

## Introduction

First identified in China in December 2019 as severe acute respiratory syndrome coronavirus 2 (SARS-CoV-2, hereinafter COVID-19), the coronavirus disease has since spread worldwide. Since the WHO (World Health Organization)'s declaration of a global Public Health Emergency of International Concern (PHEIC) on January 30, which was followed by a pandemic statement on March 11, many countries have implemented policy measures such as blockades of borders or social distancing^1^. As of January 30, 2021, cumulative statistics reached over 102.1 million reported cases and over 2.2 million deaths globally since the start of the pandemic^2^. By continent, based on the cumulative number of confirmed cases, as 44% of global confirmed cases occurred in the Americas, followed by 34% in Europe and 13% in South-East Asia (including South Korea). The order of these damages was the same for the cumulative deaths^2^.

In the course of the worldwide spread of COVID-19, young people, who tend to be vigorously active, have drawn criticism as actors supposedly responsible for the spread of COVID-19^3^ or have been targeted with warnings regarding their presumably indifferent behavior^4–6^. In South Korea, the region targeted in this study, when a cluster of infections occurred in an entertainment establishment in May 2020, an inter-generational conflict appeared to emerge, with criticisms of “the irresponsible and inconsiderate behavior of young people^7^.” More recently, concerns about young people have been intensifying as the public's interest in COVID-19 declined^8,9^, due to a phenomenon that has been called “pandemic fatigue^10^.” These criticisms of young people's behavior were not entirely unfounded. To review research on young people's responses to COVID-19, Hutchins et al. conducted a COVID-19 impact survey and analyzed age-specific characteristics in self-reported mitigation behaviors. According to the results mitigation behaviors (mask wearing, handwashing, physical distancing, crowd and restaurant avoidance, and cancellation of social activities) differed significantly by age group among adults in the United States^11^. From April to June 2020, the level of mitigation behaviors was the lowest among those 18–29 years old, and the highest among those over 60 years old. This study argued that there is a need for improving communication and adjusting policy priorities to encourage mitigation behaviors in young people^11^. According to the analysis of the use of digital contact tracing apps in Germany, the group over 50 years old showed higher uptake rates than the young people^12^. Nivette et al. found that, contrary to expectations, non-compliance with quarantine measures was more frequent among men, highly educated persons and non-immigrants, based on a cohort sample of young people interviewed in Switzerland^13^. Non-compliance was shown to be especially high among young adults with high indicators of “antisocial potential” such as legal cynicism or low confidence in the government.

These criticisms and concerns about young people's awareness and behaviors during the COVID-19 pandemic may seem justified. However, this study poses the question of whether it is appropriate to criticize people's behavior based on such generational distinctions, in a situation where the spread of COVID-19 has continued so long that it has now given rise to concerns about pandemic fatigue. The key question this study explores is "Did young people behave indifferently or in a manner distinct from other generations in response to COVID-19, during the global COVID-19 pandemic?" We propose a scientific method for analyzing assertions or assessments about young people in response to COVID-19, deriving insights that will help formulate appropriate policies and social evaluations of them. We expect that these implications of the study will help reduce unnecessary generational conflict and prepare for post COVID-19 recovery in consideration of the characteristics of each generation.

## Methods

### Hypotheses and research structure

The research question can be investigated by testing the following research hypotheses: that “young people's awareness of COVID-19 is different from other generations (H1)”^11,14–17^ and that “young people's (consumption) activities caused by COVID-19 is different from other generations (H2)^18,19^." To answer the above research question and verify our hypotheses, we constructed our research model as follows. First, our key variables, awareness and consumption behavior, were operationally defined as internet information searches^1^ and credit card use^20^, which were respectively measured by search traffic and the scale of credit card usage. To analyze differences in the level of interest in COVID-19 demonstrated by young people, we compared the changes in internet searches in correlation to new confirmed cases of COVID-19 to changes in the reference group. To analyze the differences in activity caused by COVID-19, we also compared young people with the reference group based on credit card use by sectors of industry. Our categorization of industries was based on the categories used by credit card companies: our study included 25 specific sub-sectors, under the five larger sector categories titled Leisure, Recreation, ICT, Daily Living, and Restaurants/Fashion. Next, we chose South Korea as our target area and set the target period as 1 year from the time of the first confirmed case (January 20, 2020 – January 20, 2021), because this made it possible for us to observe three major wave sections of outbreaks that can be broadly distinguished from one another. Lastly, we defined generations based on age (age group generation). *Young people* was defined as those from 20 to 29 years old (twenties) and the *reference group* was set as those in their fifties (50–59 years old). Table 1 summarizes the data collection period and the age range of young people. In South Korea, the 50s age group had the highest proportion of new confirmed cases, and this group also constituted the highest proportion of the population at 16.4% (while the 20s age group comprised 13.4% of the population)^21^. For this reason, the 50s were set as the reference group (see Supplementary Table T1).

**Table 1:** Key research variables.

Variable	Description	Data collection period	Young people’s age range	Source	Hypothesis
Internet searches	Relative Search Volume for “Corona” (in Korean) Search Trends	Jan. 20, 2020–Jan. 20, 2021 (daily)	19–29 years olds	Naver DataLab	Hypothesis 1
New cases	New confirmed cases of COVID-19	Jan. 20, 2020–Jan. 20, 2021 (daily)	20–29 years olds	Korea Disease Control and Prevention Agency (KDCA)	Hypothesis 1
Credit card use	Relative credit card usage volume by sub-sector	Oct. 2019–Dec. 2020 (monthly)	20–29 years olds	BC Card	Hypothesis 2

### Data collection

As for data collection, the search data we used consisted of daily statistics provided by the Korean search engine *Naver*. In South Korea, Naver has a much higher share of the search engine market than *Google* (58.9% vs. 33.0%)^22^. Here, we collected search information from both generations via Naver DataLab, and since the data was collected by generation, all search data was collected as daily relative search volume (RSV), that is, a normalized value based on the search volume on the day with the highest domestic search volume during the data collection period (with its value set as 100)^23,24^. Descriptive statistics for the variables described in Table 1 were report in Supplementary Table T2. Our data on new confirmed cases consisted of the daily South Korean confirmed cases statistics provided by the Korea Disease Control and Prevention Agency (KDCA)^25^. A detailed description of the new cases of COVID-19 and the search data in South Korea were reported in Supplementary Figure F1. Credit card use for testing the second hypothesis was measured by the monthly data on number of credit card uses by business sector provided by *BC Card*, and as above, the maximum monthly value for each age group during the analysis period was standardized to 100. BC card, which provided the credit card usage information analyzed in this study, has a market share of 16% in South Korea based on transaction amounts from 2017 to 2019 and is the second highest performing credit card company^26^. BC Card provided data on usage classified by age group and by type of industry, although limited in scope. The source of our data was the publicly available data released by Naver DataLab^27^. Since the released data consisted of monthly usage aggregate statistics, it was not possible to identify individual credit card users. In the target country, South Korea, the statistics on the usage of different methods of payment are as follows: based on the total number of transactions, 43.7% of transactions were made by credit cards, which made up the highest percentage, followed by 26.4% by cash and 19.2% by debit/debit cards^28^. In this study, we limited the regional area of credit card merchants to Seoul (over 50% share), due to the limited scope of data^29^. A detailed description of credit card usage were reported in Supplementary Figure F2.

### Hypothesis testing methods and data conversion

The analysis methods we used to test the first hypothesis regarding young people's awareness were VAR (Vector Autoregression) analysis and Granger causality analysis, chosen from among various time series analysis methods^30,31^. We analyzed the relationship between new confirmed COVID-19 cases and searches for COVID-19 information in all of the search data, and we verified whether it was possible to observe a weakening of awareness in this correlation. Furthermore, we examined whether the differences in searches by age group were statistically significant. We analyzed the correlation between searches and new (confirmed) cases, statistically analyzing the relationship using the methods of cross-correlation, Granger causality, and VAR analysis^23,32,33^. Prior to developing the VAR model, we tested whether the variables (searches and new cases) were stationary time series by conducting a unit root test. For both variables, the Dicker–Fuller GLS (ERS) test showed there was no unit root at a 5% significance level^34,35^. For the first hypothesis, we analyzed daily data and the target period for analysis ranged from January 20, 2020, when the first confirmed case was detected, to January 20, 2021 as shown in Table 1. To empirically verify the phenomenon of weakening awareness, we divided the analysis target period into three wave sections, reflecting the trends in the spread of COVID-19 (see Supplementary Figure F1). In each wave, the peak and new confirmed cases were 909 on February 29, 441 on August 26, and 1237 on December 24. Sections were divided as shown in Table 2 so as to distribute the data evenly among the sections and reflect the timing of the peak in the 2nd and 3rd waves.

**Table 2:** Division of periods of COVID-19 spread, for the empirical verification of weakened awareness.

Category name	Section		
	1st Wave	2nd Wave	3rd Wave
Peak day	February 29, 2020	August 26, 2020	December 24, 2020
Period	2020 1/20–2020 7/19	2020 7/20–2020 10/19	2020 10/20–2021 1/20
Observations	182 days	92 days	93 days

For the second hypothesis, we conducted both the paired t-test and the Wilcoxon test to analyze young people's consumption activities. The size of credit card usage and monthly trends were compared to see the degree to which consumption recovered in the 25 sub-sectors categorized into five sectors, specifically among consumers in their twenties and fifties. Our base of comparison was the monthly credit card consumption in each age group in the 4th quarter of 2019, before the outbreak of COVID-19.

To explain our data conversion process more specifically, direct comparisons of young people and the reference group by credit card use would not have yielded very significant results in this study due to differences in income, etc. Therefore, instead of directly using the collected credit card usage data shown in Table 1, in this study, the level of credit card usage in each age group was converted to express the extent of relative recovery for the purpose of comparison. In other words, our study focused on comparing the generations based on the relative level of recovery of credit card use over time (before and after the COVID-19 outbreak). The comparison was based on the average credit card usage of each group in the fourth quarter of 2019. By dividing the monthly credit card usage level with this value, we calculated the “*credit card usage intensity*” by sub-sector, month, and age group. In this study, since monthly credit card usage data were compared by generation, there was no independence, and although the monthly average was used, the sample size was too small to assume parametric distribution. Therefore, instead of performing only the paired t-test, we simultaneously performed the Wilcoxon (signed rank) test and presented these results together^36,37^. Before using the paired t-test, we verified whether our monthly card usage data for the 5 sectors and 25 sub-sectors violated the statistical assumption of “independence across elements,” and whether autocorrelation was present through ACF (Autocorrelation Function)^38^.

## Results

### Generational differences in the awareness of COVID-19

**Figure 1:**
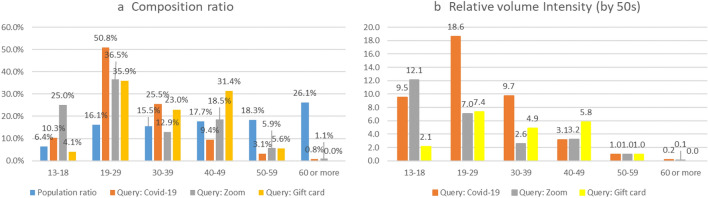
Comparison of search volume distribution by age for key search queries.

Figure 1a shows the percentage occupied by each age group in the searches performed by the public for information on COVID-19. Although the youth group was smaller than the reference group in terms of population or confirmed cases (see Supplementary Table T1), as seen in Fig. 1a, the percentage of young people (19–29 years old) was overwhelmingly high (50.8%). To compare the differences in relative search volumes (RSV), taking account of the size of the population, we divided the ratio of the total search volume by the proportion of the search population aged 13 or older and then converted the scale based on age 50s: the values thus obtained were defined as “*relative volume intensity*” and is shown in Fig. 1b.

The relative search intensity of young people on the subject of COVID-19 was 18.63; this level of interest was 18 times higher compared to the reference group (50s), when considering their proportion of the population. Since this difference in search volume may reflect generational differences in information search tendencies or the distribution of Naver users, we also compared other search queries, as presented in Fig. 1b. First, we examined the distribution of searches by age for the online platform “Zoom,” which gained attention as remote work and online classes became more commonplace due to COVID-19^39,40^. Users aged 13–18 showed the highest relative search intensity at 12.1, which was to be expected due to the widespread use of Zoom for educational purposes. The intensity of young people was 7.04, higher than that of the reference group, but significantly lower than the intensity of searches for COVID-19. Next, we looked at searches for “cultural gift certificate,” which ranked first among popular search terms in the sectors of “Leisure” and “Daily Life” in the past year^41^. In this case, the intensity among young people was 7.35, similar to the level for Zoom. Young people thus exhibited a higher interest in COVID-19 than in an online platform that had rapidly increased in usage in schools and workplaces or in the usual top-rated search term, which suggests that young people have never been indifferent to COVID-19. This confirmed that the generation in their twenties exhibited a strong interest in COVID-19.

**Figure 2:**
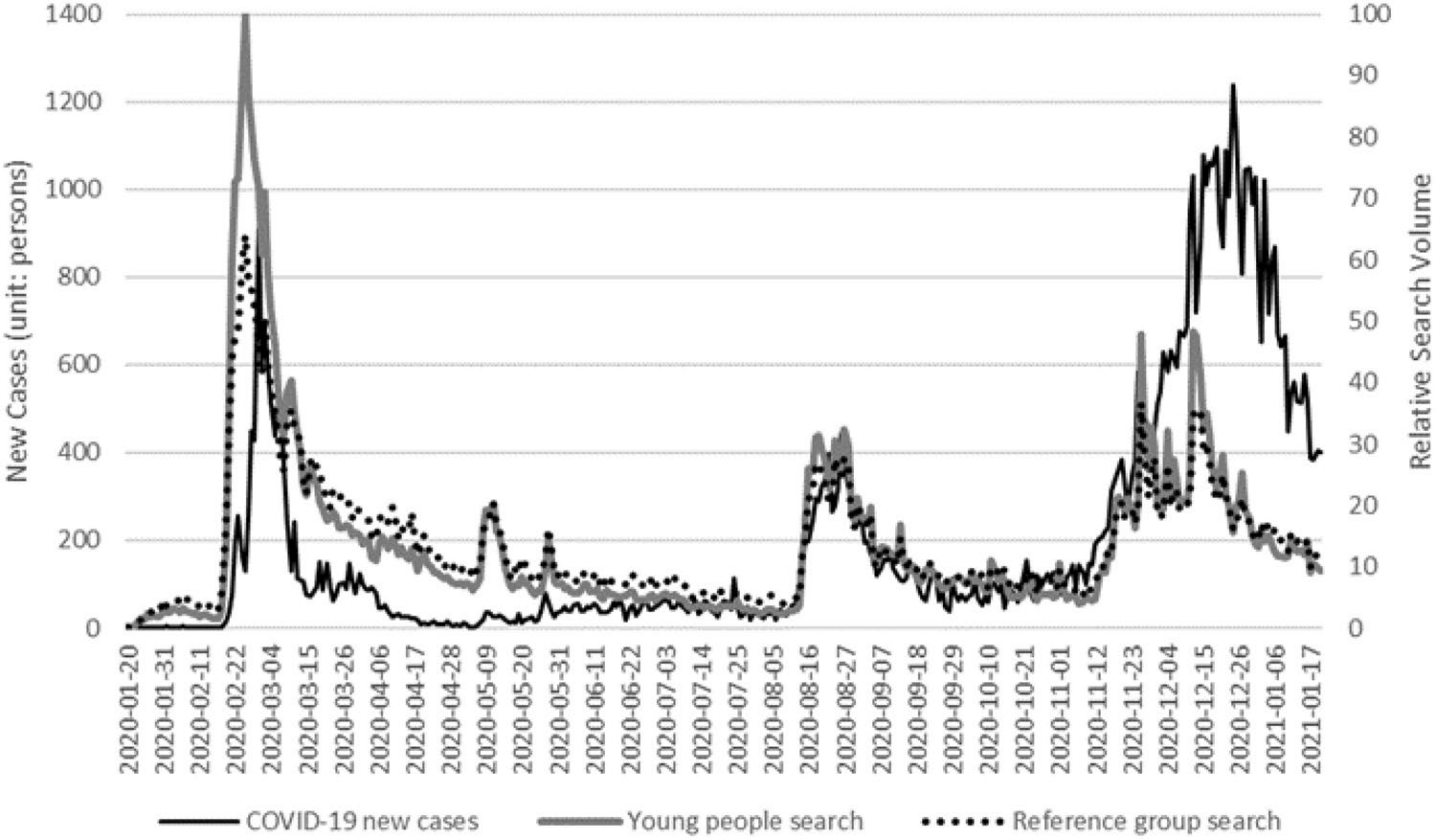
Comparison of search trends between young people and the reference group.

In addition to the magnitude of interest, Fig. 2 shows the temporal changes in interest. In Fig. 2, to compare the search traffic standardized to the highest value, the reference group was modified, with the total search area matched to young people. Figure 2 shows that the flows of these two standardized groups, are almost identical. In cross-correlation, the correlation coefficient was the highest when there was no lag, at 0.98 (see Supplementary Table T3). Even in Pairwise Granger causality tests in Table 3, Granger causality was hardly observable. However, in lags 2 and 3, the null hypothesis that young people's searches did not precede the interest of the reference group was rejected at a significance level of 5%, which confirmed that the interest of young people can have Granger causality preceding the reference group by around 2–3 days. Even when we observed the changes over time, there were hardly any differences between those in their twenties and fifties.

**Table 3:** Pairwise Granger causality tests results between groups for all sections.

Null hypothesis	Statistic					
	Lag 2		Lag 3		Lag 4	
	F-Statistic	Prob	F-Statistic	Prob	F-Statistic	Prob
Reference group does not cause young people	1.061	0.347	0.696	0.555	0.578	0.679
Young people do not cause reference group	3.776*	0.024	3.640*	0.013	1.904	0.109

Here *p < 0.05.

Finally, we examined whether there were differences between young people and the reference group by section. Examining each section in Table 4, we noted that there was little difference between groups in the 1st wave section; rather, it appears that young people responded faster than the reference group in the 2nd wave section. Even in the 3rd wave section, young people’s responses did not fall behind those of the reference group. Therefore, there was no basis for judging that the interest of young people lagged behind the reference group in any section.

**Table 4:** Pairwise Granger causality tests results between groups by section.

Category name	Section					
	1st Wave		2nd Wave		3rd Wave	
	F-Statistic	Prob	F-Statistic	Prob	F-Statistic	Prob
**Lag 2**						
Reference group does not cause young people	1.672	0.191	2.775	0.068	6.858**	0.002
Young people do not cause reference group	0.794	0.454	5.787**	0.004	6.992**	0.002
**Lag 4**						
Reference group does not cause young people	3.585**	0.008	3.229*	0.017	2.371	0.059
Young people do not cause reference group	3.591**	0.008	4.667**	0.002	2.662*	0.039

Here *p < 0.05; **p < 0.01.

To synthesize the above results, although there was a decline in interest in COVID-19 in South Korea, which is the cause of pandemic fatigue, in this process, young people showed a rather high level (magnitude) of interest compared to the reference group, and their response was not delayed compared to the reference group. Therefore, we judged that the first hypothesis that “young people's awareness of COVID-19 is different from other generations,” that is, lower than that of other generations, could be rejected. If such a hypothesis were to be adopted, it would need to be revised to mean that the interest of young people was higher.

### Generational differences in behavior caused by COVID-19

In most cases, the intensity of monthly card use in the reference group was higher than the intensity among young people. A detailed monthly credit card usage by age group were reported in Supplementary Figure F3. Table 5 presents the results of statistical analyzing the mean and statistical differences in the trends sector and age group (see Supplementary Figure F3). According to Table 5, the mean difference between Leisure and Recreation was not statistically significant in either parametric or nonparametric analysis. By contrast, the other three sectors (ICT, Daily Living, Restaurant/Fashion). We noted, however, that despite the relatively low income of people in their twenties, their consumption was not small in the sectors of Leisure and Recreation.

**Table 5:** Credit card use intensity average and statistical test result by sector and group.

Sector	Young people monthly average (%)	Reference group monthly average (%)	Significance	
			Wilcoxon test	Paired t test
Leisure	70.2	69.2	1.000	0.728
Recreation	62.5	68.4	0.084	0.055
ICT	91.2	102.5**	0.003	< 0.001
Daily living	74.0	83.5**	0.002	< 0.001
Restaurant/fashion	72.5	81.2**	0.002	< 0.001

Here * is based on the non-parametric Wilcoxon test result, and is indicated at a significantly higher monthly average (**p < 0.01).

We compared young people and the reference group by specific sub-sector of industry. As shown in Table 6, in the Leisure sector, only two sub-sectors showed significant differences: there was statistically higher monthly credit card use intensity among young people for tourist accommodation and in the reference group for golf courses. Specific monthly credit card use intensities corresponding to sub-sectors and age groups were reported in Supplementary Figure F4. Table 6 shows that in the Recreation sector, only two sub-sectors had significant differences. Statistically higher monthly credit card use intensity was shown among young people for entertainment and in the reference group for cultural facilities. Table 6 also presents detailed sub-sector comparison results for the three sectors (ICT, Daily Living, Restaurant/Fashion) that showed statistically significant differences in Table 5. These are sectors in which, as seen in Table 6, young people had significantly lower monthly credit card use intensity than the reference group, and likewise, most of the detailed sub-sectors showed the same results. Only in the case of public transportation, which belongs to the Daily Living sector, did young people show significantly higher credit card use intensity than the reference group. Looking at each sector, first, we found that the three sub-sectors within the ICT sector showed significant differences: all young people had monthly credit card use intensity that was lower to a statistically significant degree than that of the reference group. Next, in the Daily Living sector, four of the specific sub-sectors with the exception of gas stations only showed significant differences. As mentioned above, only public transportation formed a contrast by showing significantly higher credit card use intensity among young people than in the reference group. Lastly, in the Restaurant/Fashion sector, all four sub-sectors with the exception of bars (pubs) showed significant differences, in that young people again had statistically significantly lower monthly credit card use intensity than the reference group.

**Table 6:** Credit card use intensity average and statistical test result by age group.

Sector and sub-sector	Young people monthly average (%)	Reference group monthly average (%)	Significance	
			Wilcoxon test	Paired t test
**Recreation**				
Movies	25.4	31.1	0.230	0.523
Companion animals	89.7	91.4	0.695	0.475
Entertainment	62.3*	51.0	0.021	0.014
Cultural facilities	50.7	75.3*	0.034	0.028
Hobbies	84.2	93.0	0.060	0.173
**Leisure**				
Golf courses	94.8	115.4*	0.019	0.016
Swimming pools	43.6	42.5	1.000	0.760
Other leisure	69.5	63.5	0.099	0.072
Tourist transportation	75.1	71.1	0.388	0.260
Tourist accommodation	67.9*	53.6	0.015	0.004
**ICT**				
IT service	63.6	82.4**	0.002	< 0.001
Communication service	90.5	105.8**	0.002	< 0.00
Mobile	73.1	91.9**	0.005	0.000
Home appliances	126.7	131.7	0.638	0.280
Media	101.9	100.6	0.638	0.722
**Daily living**				
Hospital	84.7	91.5*	0.010	0.008
Gas station	73.9	78.1	0.182	0.132
Public transportation	62.7*	55.5	0.013	0.002
Food	75.0	105.8**	0.002	< 0.001
Interior design	73.8	86.4**	0.002	< 0.001
**Restaurant/fashion**				
Korean food	64.1	72.4*	0.010	0.003
Bar (pub)	67.1	65.0	0.374	0.311
Cafes	76.3	96.2**	0.002	< 0.001
Fashion	70.9	79.0*	0.019	0.018
Beauty parlor	84.0	93.3**	0.002	< 0.001

Here * is based on the non-parametric Wilcoxon test result and is indicated at a significantly higher monthly average (*p < 0.05; **p < 0.01).

Putting the above results together, the results of the paired t-test and the Wilcoxon test comparing credit card usage trends during the COVID-19 period indicated that out of the 25 specific sub-sectors, only 3 showed significantly higher consumption recovery by those in their twenties than those in their fifties, namely “tourist accommodation” (classified under the Leisure sector), “entertainment” (in the Recreation sector), and “public transportation” (in the Daily Living sector). On the other hand, the reference group significantly more in 12 sub-sectors such as golf courses, cafes, and food, and there was no inter-generational difference in 10 sub-sectors, such as movies, companion animals, and hobbies. Therefore, the second hypothesis, that “young people's (consumption) activities caused by COVID-19 is different from other generations,” was partially adopted, but it could not be concluded that young people engaged in more activity.

## Discussion

According to previous studies, young people showed lower participation in mitigation behaviors than other age groups^11^, and non-compliance with quarantine measures was more frequent^12,13^. Based on these facts, young people have been criticized not only in South Korea but in many other countries^3–9^. However, in this study, we could not find any evidence that the younger generation in South Korea was significantly more insensitive to COVID-19 and they did not exhibit a more rapid decrease in awareness compared to reference group. In analyzing consumption activities, we confirmed that young people were relatively inactive due to their low income^42^. We argue the following based on these findings.

First, we may need to reassess the role of young people in the dissemination of COVID-19 or weakening of awareness as shown in Fig. 2. Through the search data, we verified the phenomenon of the public's interest in COVID-19 gradually weakening, but also found that young people had sustained a high level of interest and continued to show quick responses. Of course, here one may question whether search volume is equivalent to interest. It is possible to interpret searches as merely an act of finding rudimentary information about COVID-19, which does not necessarily indicate interest. However, in the searches for COVID-19, queries that included the phrase “what is/are” were concentrated only in the early stage^43^. Despite the extended duration of the COVID-19 pandemic, searches for COVID-19 continued for one year and this can be regarded as sufficient evidence of interest. As explained above, the decline in interest could also be seen as caused by a decline in general simple information retrieval^43^. or the elimination of the hype phenomenon (excessive interest in new risks)^44^, but considering the decrease in search volume over several months in Fig. 2, we judged that it was appropriate to explain this phenomenon as fatigue^8^. In South Korea, the fact that young people's interest in COVID-19 was particularly high, and social activity (consumption) declined due to quarantine measures may be related to social norms, as in the case of Japan. Once a consensus had developed in public opinion that going out in an emergency situation constitutes anti-social behavior, violating these norms would incur social stigma that increased psychological costs^45^. Regardless of the effects of factors such as relatively low income or social stigma, the high interest and restraint of activities shown by young South Koreans even in a situation where public interest was decreasing contributed to the outcome that their infection rate was similar to that of other age groups and that there was no sudden increase in infection among young people as in the case of other countries. As of February 1, 2021, the cumulative number of confirmed cases per million people in South Korea was 1,538, which was low compared to 79,519 cases in the U.S. and low even compared to 3,099 cases in Japan, which is geographically closer. When the cumulative confirmed cases were classified by age and generation, in the United States, the number of cases among young adults (18–23 years old) was especially higher than other age groups^17^, but in South Korea, the cumulative confirmed cases were highest among those in their fifties, and groups with the second highest number were those in their twenties and in their sixties (see Supplementary Table T1)^25^.

Second, in responding to COVID-19, there may be differences by generation in terms of behavior rather than awareness, and therefore when seeking to strengthen current public quarantine defense measures for a specific industry, it may be necessary to target the policy (through promotion) to a specific generation rather than trying to strengthen the awareness (or attention) of the general public, and we should be aware that such industry-specific policies may cause different side-effects depending on the generation. The South Korean government only implemented social distancing measures with adjustments to reflect severity and did not impose a full lockdown. Therefore, the data on credit card consumption activity by industry sector mainly reflects the effects of social distancing and in some sections, there were limited effects caused by the Disaster Relief Fund. As shown in Supplementary Figure F3, the effect of social distancing was prominently notable in the Leisure and Recreation sectors, and the effect of Disaster Relief was concentrated in the Daily Living and Restaurant sectors. Since many instances of the relative decline in young people's activities were found in these sectors, we may judge that they actively complied with policy. This finding diverges from the conclusions of other preceding studies: in South Korea, we found that credit card usage did not indicate young people failed to follow policy measures or exhibited passive participation in mitigation behaviors^11,13^. In Tables 5 and 6, in almost all sectors and sub-sectors, the reference group showed a faster recovery in consumption activity than young people (Supplementary Figure F2). This result was expected and considering that young people's income on a household basis is less than half that of the reference group ($32,156 vs. $68,708)^46^ and as explained in Supplementary Table T1 and that young people's economic activity declined relatively more^42^, we can attribute this result to the effect of disparities in disposable income^18^.

Third, such inter-generational behavioral differences can be a very important variable in the impact of COVID-19 and related predictions. Our study did in fact confirm that in the entertainment sub-sector, in which consumption by those in their twenties was significant, this young generation was indeed leading the phenomenon of “return to experience and hedonism, with a caveat” discussed by Zwanka; meanwhile, “stock up mentality and online ordering” was confirmed to be led by the reference group (in the food industry, classified under Daily Living sector)^39^. Even though this is a long-term, trans-generational change caused by COVID-19, there may be a specific generation leading the onset of change, and one generation may not be clearly perceive changes driven by another generation. To analyze such changes thoroughly, the characteristics of the specific age group must be adequately considered. For example, young people's use of tourist accommodations may on the surface seem to be related to the phenomenon of “virtual reality replacing travel” but our research reveals that it may be more related to the phenomenon of “return to experience and hedonism, with a caveat^39^.” Young people are also under great psychological stress from COVID-19^47^, and there is a need to be more understanding towards the fact that young people may take actions to relieve stress in certain sectors of Recreation and Leisure. Above, in our presentation of our findings and our discussion, we emphasized that the responses of young people to COVID-19 were not different from other generations and in some respects young people were more active in their responses, but here we further argue that in predicting change, an approach based on generational differences can contribute to more sophisticated predictions. For the purpose of identifying sectors of industry where there were differences between generation, it is of limited use to directly compare the relative level of credit card usage recovery by generation. Young people suffered greater economic hardships from COVID-19, and therefore it is natural that credit card usage recovery would be slower than that of the reference group and it is necessary to take account of this phenomenon while examining the differences between the two groups. Supplementary Table T5 provides the results of Bayesian inference analysis assuming a prior distribution (distribution subject to hypothesis testing) of credit card use recovery of age groups by sector^48^. According to the Bayesian inference results, the sub-sectors with significantly higher consumption by young people included media and bars (pubs) in addition to tourist accommodation, entertainment, and public transportation while the sub-sectors in which the reference group was significant were golf courses, food, and cafes. Because it is possible that different generations will lead changes in these sub-sectors in the future, it would be especially useful to pay attention to the specific movements of the leading generation for the purpose of monitoring future changes or predictions.

As summarized above, our study aimed to help decision making on appropriate policies or social judgment, by objectively analyzing generational differences in reactions or awareness of COVID-19 in a society where COVID-19 is spreading. We endeavored to provide insights on the differences in awareness and behaviors by generation, classified by sub-sectors of industry, to better respond to future social changes following COVID-19. Of course, there will be limitations in generalizing the case of one country, but there is no denying that the active participation (attention and behavior) of young people has also contributed to the successful quarantine in South Korea. By revealing these findings, this study hopes to be of little help in resolving unnecessary inter-generational conflicts in COVID-19. A person from any generation can be cynical or non-compliant with government quarantine policy^13^. There may be many such people in the younger generation, but this study shows that it would not be appropriate to criticize the entire generation. Our study aimed to show that such a generation-centered approach may not be of much help in overcoming COVID-19, by analyzing the cases of young South Koreans. This departure from a generation-centered approach is what differentiates this study the most, and he strength of the study is that it supported our argument through quantitative model analysis. We were wary of the type of selective critical perspective that underscores the connection between a generation and the characteristics of specific sectors of industry or places only in some cases. This does not mean that this study argues that the few places where younger people have been more likely to gather are not dangerous; the study explains that young people have shown stronger interest in COVID-19 than the reference group, and that contrary to general perception, there have been far fewer places or sectors in which young people have been more active.

Despite the differences of these studies, this study has limitations. One of the limitations of this study is that since analyzed one specific country, it may not be possible to draw broader generalizations. In our statistical analysis of monthly credit card consumption activity, both parametric and non-parametric methods were presented, but the number of cases analyzed (12 months) remain insufficient. However, the paired data used in our statistics are relatively robust, as it consisted of monthly average data, not merely a single event, and therefore we believe there will be no significant negative effect on the stability of this study’s results. It should also be noted that since we relied on public data, only a limited range of data was accessible, and it was not possible to present the change in monthly card consumption compared to the previous year. It is regretful that due to this limitation, this study could not address how seasonal factors may affect the interpretation of the effect of governmental relief funds. Once these limitations are overcome to expand the scope of this study, it will be possible to further generalize this study’s findings and contribute more to performing objective assessments of young people in COVID-19 or formulating related policies.

## Supplementary Information

Supplementary Information.

## Data Availability

The sources of data included in this study were Naver DataLab for search information (https://datalab.naver.com/keyword/trendResult.naver) and credit card usage information (https://datalab.naver.com/local/card.naver).
