# Supplementary material for: Young people are not blameworthy: the generation’s awareness of COVID-19 and behavioral responses
Source: Sci Rep. 2021 Dec 8;11:23595. doi: 10.1038/s41598-021-03036-x (PMC8654866; doi:10.1038/s41598-021-03036-x)
Supplement: Supplementary file 1 — Supplementary Information. [file 41598_2021_3036_MOESM1_ESM.pdf]

Young people are not blameworthy: the generation's awareness of COVID-19 and behavioral responses

Seung-Pyo Jun[1*], Hyoung Sun Yoo[1], and Chul Lee[2]

TABLE T1. COVID-19 NEW CONFIRMED CASES AND POPULATION DISTRIBUTION

Table T1 shows the cumulative confirmed cases and presents the search population composition ratio, to explain differences by age in the total search volume. Table T1 also provides data that suggests the economic hardships young people experienced during the COVID-19 period. Compared to January 2020, the share of the economically active population in all age groups had decreased as of January 2021. However, whereas in the reference group the share decreased by only 1.8% compared to the previous year (1.4%p), it decreased among young people by 5.4% (3.4%p), from 62.8% to 59.4%, confirming that the younger a person was, the greater the impact of the pandemic was on economic activities.[4]

Table T1. Current status of cumulative confirmed cases by age (February 19, 2021) and population distribution (2019)

Age group	Cases (%)	Deaths (%)	Lethality	Population (%)	Search population ratio	Economic activity participation rate (2020. Jan. vs. 2021. Jan.)
80 or more	4,259 (4.9)	881 (56.7)	20.7%	2,530,970 (4.8)	5.4%	-
70-79	6,609 (7.6)	423 (27.2)	6.4%	3,521,769 (6.7)	7.5%	-
60-69	13,579 (15.7)	179 (11.5)	1.3%	6,181,640 (11.8)	13.2%	41.5% : 40.8%++
50-59	16,151 (18.7)	51 (3.3)	0.3%	8,608,070 (16.4)	18.3%	76.7%: 75.3%
40-49	12,432 (14.4)	12 (0.8)	0.1%	8,304,629 (15.8)	17.7%	79.7%: 78.4%
30-39	11,218 (13.0)	6 (0.4)	0.1%	7,304,423 (13.9)	15.5%	78.9%: 77.8%
20-29	13,063 (15.1)	1 (0.1)	0.0%	7,026,161 (13.4)	16.1%*	62.8%: 59.4%
10-19	5,800 (6.7)	0 (0.0)	-	4,871,356 (9.3)	6.4%**	9.1%: 7.2%+
0-9	3,465 (4.0)	0 (0.0)	-	4,158,165 (7.9)	0.0%	-

Source: KDCA[2] & KOSIS[4], Note: * young people 19~29 years old, * * 13-18 years old, + 15-19 years old, ++ Over 60 years old, The fatality rate is the number of deaths/the number of confirmed cases ∗ 100

[1] Korea Institute of Science and Technology Information and Science & Technology Management & Policy, University of Science & Technology (UST), [2] Korea Institute of Science and Technology Information, corresponding author e-mail: spjun@kisti.re.kr; spjunn@msn.com

TABLE T2. DESCRIPTIVE STATISTICS

Table T2 shows descriptive statistics for the raw data on detailed variables related to the 3 variables explained in Table T2.

Table T2. Descriptive statistics of raw data of variables used in research

Variable		N	Min.	Max.	Mean	S.D.	skewness	kurtosis
New (confirmed) cases		367	0.0	1237.0	201.4	274.857	1.869	2.685
Search	All	367	0.1	100.0	15.2	14.948	2.622	8.681
	Young people	367	0.1	100.0	14.1	14.921	2.754	9.423
	Reference group	367	0.1	64.0	14.0	10.352	1.844	4.419
Leisure sector	Golf courses	15	4.9	100.0	38.2	35.270	0.913	-0.828
	Swimming pools	15	2.0	100.0	24.5	28.785	1.632	2.169
	Other leisure	15	73.0	100.0	87.2	6.224	-0.147	1.577
	Tourist transportation	15	77.6	100.0	88.1	7.069	0.429	-0.994
	Tourist accommodation	15	0.0	100.0	63.8	28.584	-1.365	1.506
Recreation sector	Movies	15	0.0	100.0	52.3	27.183	-0.375	0.118
	Companion animals	15	16.0	100.0	43.7	27.555	1.046	-0.238
	Entertainment	15	49.0	100.0	68.5	15.222	1.112	0.240
	Cultural facilities	15	36.0	100.0	48.1	15.827	2.841	9.111
	Hobbies	15	54.0	100.0	68.9	11.354	1.498	3.016
ICT sector	IT service	15	16.0	100.0	57.3	25.340	-0.215	-0.911
	Communication service	15	27.0	100.0	64.0	21.752	-0.297	-0.647
	Mobile	15	2.4	100.0	42.4	31.682	0.434	-1.125
	Home appliances	15	6.0	100.0	49.3	31.986	0.351	-1.024
	Media	15	21.0	100.0	67.3	23.005	-0.552	-0.267
Daily living sector	Hospital	15	22.0	100.0	57.6	21.434	0.390	-0.296
	Gas station	15	46.6	100.0	66.8	16.545	0.806	-0.451
	Public transportation	15	44.6	100.0	73.6	19.591	-0.165	-1.368
	Food	15	45.0	100.0	64.9	15.503	0.741	0.121
	Interior design	15	26.0	100.0	53.3	22.594	0.879	-0.153
Restaurant / Fashion sector	Korean food	15	46.1	100.0	65.9	17.498	1.170	0.254
	Bar (pub)	15	65.8	100.0	79.6	10.988	0.648	-0.696
	Cafes	15	76.6	100.0	89.0	6.319	0.117	-0.217
	Fashion	15	90.0	100.0	96.6	3.312	-1.267	0.385
	Beauty parlor	15	54.0	100.0	75.3	14.831	0.220	-1.278

FIGURE F1. COVID-19 NEW CONFIRMED CASES AND SEARCHES IN SOUTH KOREA

Figure F1 shows the similarity in the trends of COVID-19 searches and new confirmed cases, and three distinct waves of surges in COVID-19 infections. It can be seen that as we approach the third wave, the search volume relatively decreases. For reference, vaccination in South Korea began on February 26, 2021, and therefore we judged that it would have had little effect on trends during the period targeted in this study.

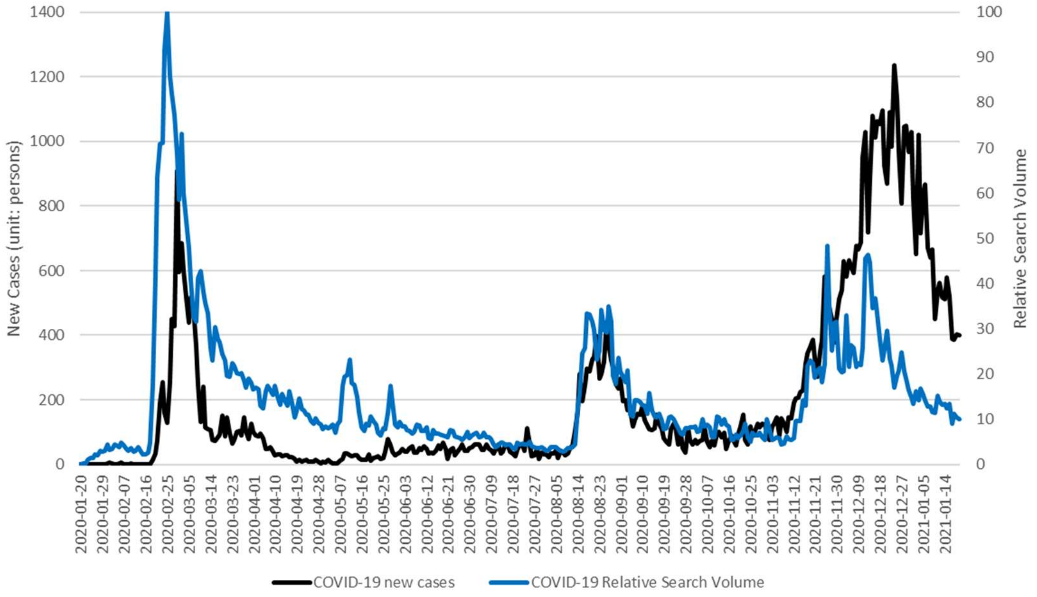

Figure F1. Comparison of new cases and COVID-19 searches

FIGURE F2. CREDIT CARD USAGE IN SOUTH KOREA

Figure F2 compares the consumption activities among young people and in the reference group for 25 sub-sectors classified under 5 broader sectors. The values shown in Figure F2 for each group is based on a comparison of credit card use cases in the 4th quarter of 2019, before the outbreak of COVID-19, and in the 4th quarter of 2020, when the third wave of COVID-19 was surging in South Korea: the value was obtained by dividing the latter by the former. This value, which we refer to as "q*uarterly recovery*," indicates the degree to which consumption activity recovered compared to 1 year ago.

As shown in Figure F2, the average quarterly recovery of credit card use among young people was lower than that of the reference group, and this was common across all five sectors. Looking at the differences across sectors, we see that in ICT there was nearly a full recovery to the level of credit card use that existed before COVID-19 (96.7%), while the recovery in the Recreation and Leisure sectors lingered at 56.9% and 60.0%, indicating that the aftereffects of COVID-19 still loomed large. Analyzed by sub-sector, the data shows that within the Recreation sector, movies showed the lowest quarterly recovery at 14%, while in the ICT sector, home appliances and media had a quarterly recovery far exceeding 100%, as consumption increased despite the spread of COVID-19.

Despite the fact that the reference group overall had higher quarterly recovery, there were sub-sectors in which young people's recovery was higher by a significant margin (a margin of 10% or more), as follows. In the Leisure sector, young people had distinctly higher quarterly recovery in tourist transportation and accommodation. In each of the other sectors, there was only one sub-sector in which young people's quarterly recovery was higher by margin of 10% or more than the previous year: entertainment under Recreation sector, media under ICT sector, public transportation under Daily Living sector and bars (pubs) under Restaurant/Fashion sector.

The quarterly recovery data discussed above helped us to intuitively judge the scale of recovery in credit card use by age group before and after the COVID-19 outbreak. However, it was difficult to draw conclusions with strong statistical significance, and another limitation was that changes in periods other than the 4th quarter could not be tracked. Therefore, in Results Section, we performed statistical comparisons of monthly trends during the COVID-19 pandemic to identify sub-sectors exhibiting significant differences.

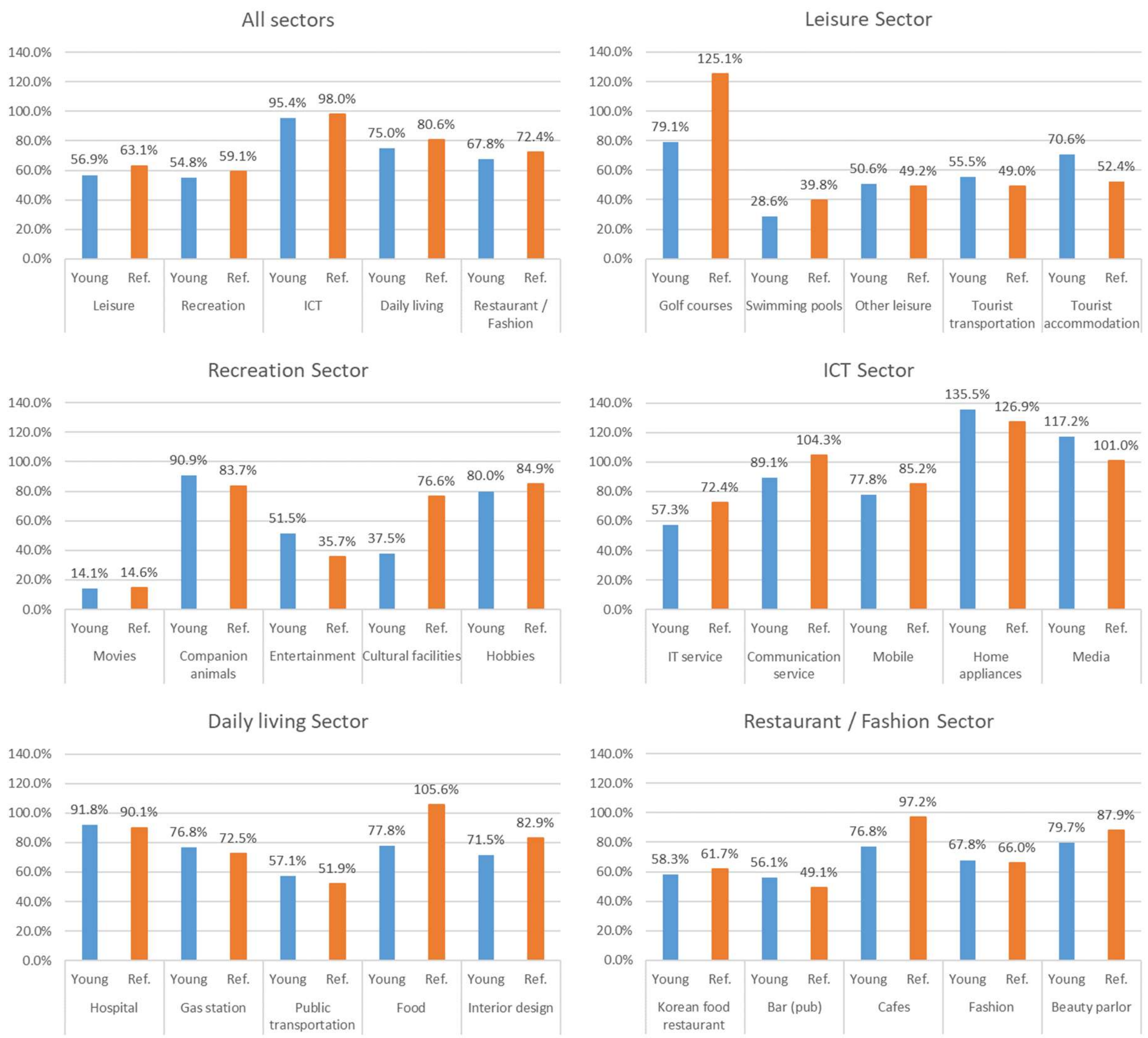

Figure F2. Comparison of quarterly recovery of credit card use before and after the COVID-19 outbreak by sub-sector

TABLE T3. THE RESULTS OF CROSS-CORELATION TEST

Table T3. Correlations are asymptotically consistent approximations

i	lag	lead
0	0.9767	0.9767
1	0.9353	0.9414
2	0.8834	0.8938
3	0.8305	0.8474
4	0.7677	0.7934
5	0.6947	0.7284
6	0.6303	0.6756
7	0.5690	0.6307
8	0.5034	0.5815
9	0.4353	0.5255
10	0.3763	0.4784
11	0.3197	0.4307
12	0.2729	0.3895
13	0.2360	0.3611
14	0.2009	0.3383
15	0.1676	0.3134
16	0.1441	0.2954
17	0.1176	0.2737
18	0.0855	0.2436
19	0.0549	0.2113
20	0.0313	0.1875
21	0.0069	0.1652
22	-0.0217	0.1375
23	-0.0426	0.1158
24	-0.0594	0.0997

FIGURE F3. MONTHLY CREDIT CARD USAGE COMPARISON BY AGE GROUP

As explained in Method Section, we used the term "*credit card use intensity*" to refer to the standardized value obtained by dividing the monthly credit card use cases in 2020 in each age group by the average value in the 4th quarter of 2019 (before COVID-19) in each age group; the monthly flow of this value is presented in Figure F3.

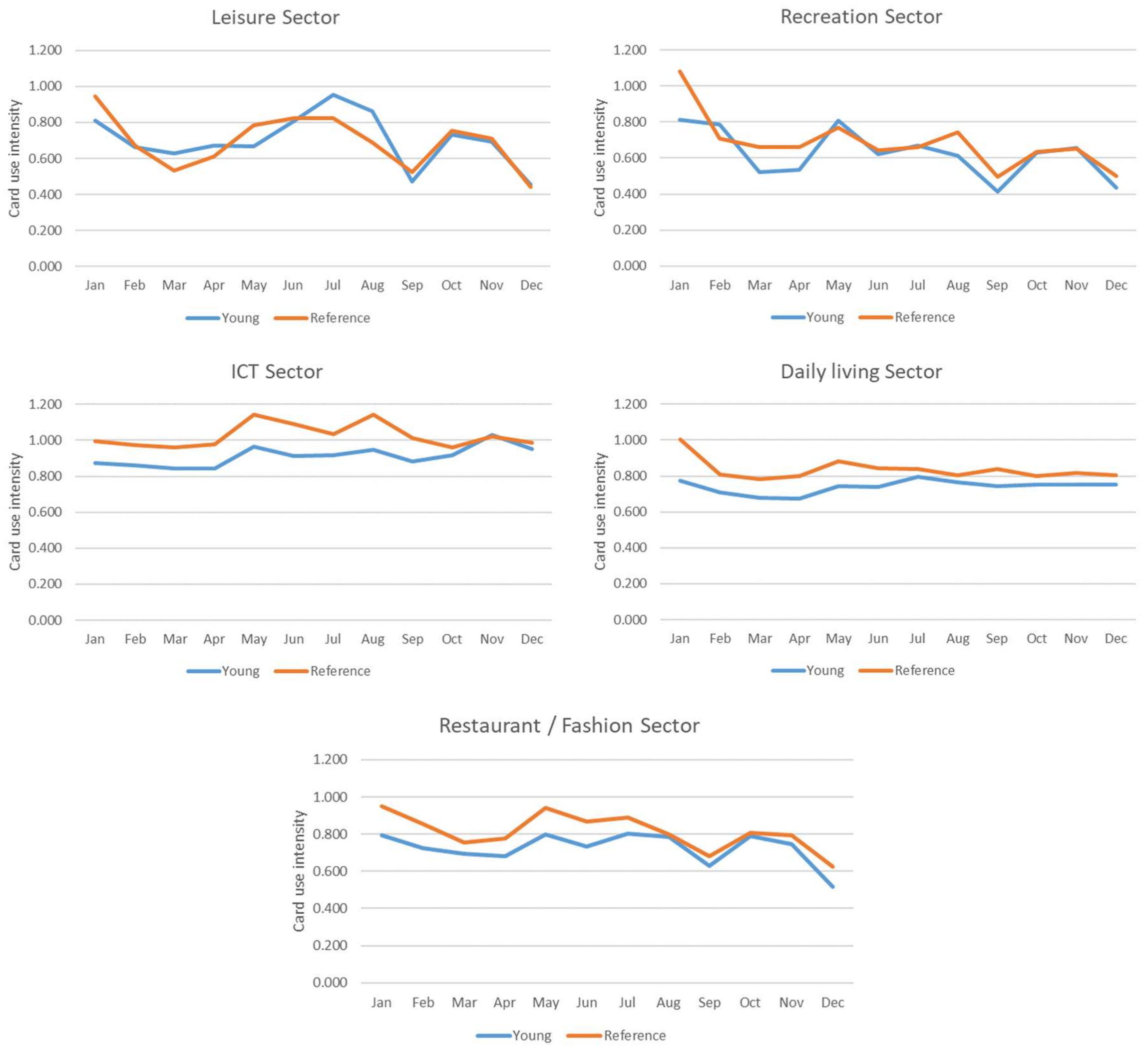

Figure F3. Credit card use intensity comparison by sector and age group

Here, the relatively high credit card use intensity does not mean that one group used credit cards more than the other groups, because this value was standardized to a different value for each group; what it means is that the group's use intensity was higher than that of the other groups compared to the 4th quarter just before the COVID-19 outbreak. Therefore, the data indicates that in the Leisure sector, young people often showed a relatively faster recovery in consumption than the reference group. In the Recreation sector, the recovery of the reference group was higher or similar to that of young people in most of the periods, and in the remaining three sectors (ICT, Daily Living, Restaurant/Fashion), the

recovery of the reference group was significantly higher than that of young people. Looking at the flow of all groups, we noted that the ICT and Daily Living sectors did not show any great change, while the remaining three sectors (Leisure, Recreation, Restaurant/Fashion) showed a slight decrease overall. Another common feature in these three sectors was the sharp and temporary decline in September: beginning on August 19th, the level of social distancing required in the Seoul metropolitan area was raised (Level 2), and finally, on August 30, a reinforced social distancing measure (Level 2.5) was implemented.[8] These events occurred in the second wave section shown in Table 2, and the peak of new confirmed cases was August 26th. As the peak of the new confirmed cases passed, the social distancing level was de-escalated on September 14th and extended to the September 27th (Level 2).[9] The impact of this series of measures is reflected clearly in the three sectors. In Figure F3, there is also one trend that is common in all sectors, although there is a difference in degree: in all sectors, credit card usage increased in May compared to April (this can be regarded as a temporary phenomenon if we exclude Leisure sector). May was the period in which the National Disaster Relief Fund, which was issued only once in South Korea, began to be distributed.[10]

FIGURE F4. COMPARISON OF MONTHLY CARD USE INTENSITY BY SUB-SECTOR AND AGE GROUP

Figure F4-1 presents a comparison of the intensity of monthly credit card use by sub-sectors within the Leisure sector and age group. Other than golf courses, these sub-sectors exhibited poor performance with a generally decreasing trend, confirming that this is an industry that has been hit relatively hard by COVID-19. The data demonstrated that the relative prospering of golf courses was led by the reference group, and in tourist accommodations, young people reduced their consumption relatively less despite the depression.

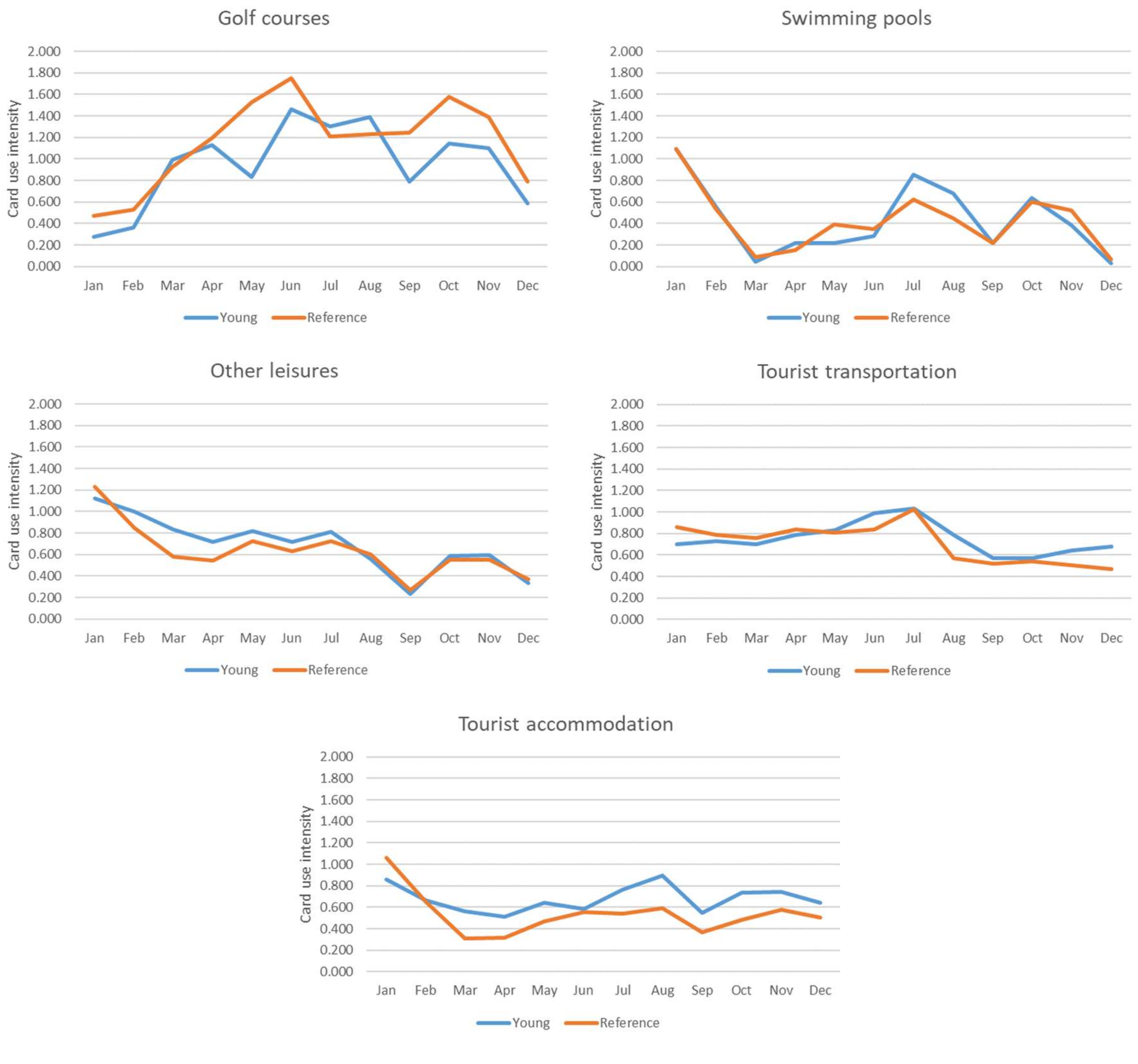

Figure F4-1. Credit card use intensity comparison by age group (sub-sectors of Leisure sector)

Figure F-2 compares the intensity of monthly credit card use by sub-sector within the Recreation sector and by age group. It shows there was a sharp decline in credit card usage in the Movies sector, and entertainment businesses and cultural facilities were unable to avert a decline. On the other hand, despite COVID-19, businesses related to companion animals and hobbies were not significantly

impacted. There was a sudden rise in credit card usage in the hobbies sector in May, when the South Korean government issued Disaster Relief Funds to the public, and this confirms that the hobbies sector was a beneficiary of that funding. Notably, this indicates that young people spent more of the relief fund in the hobbies sector. In addition, Figure F4-2 shows that there were significant differences in the entertainment and cultural facilities sectors, which were supported by consumption by different age groups.

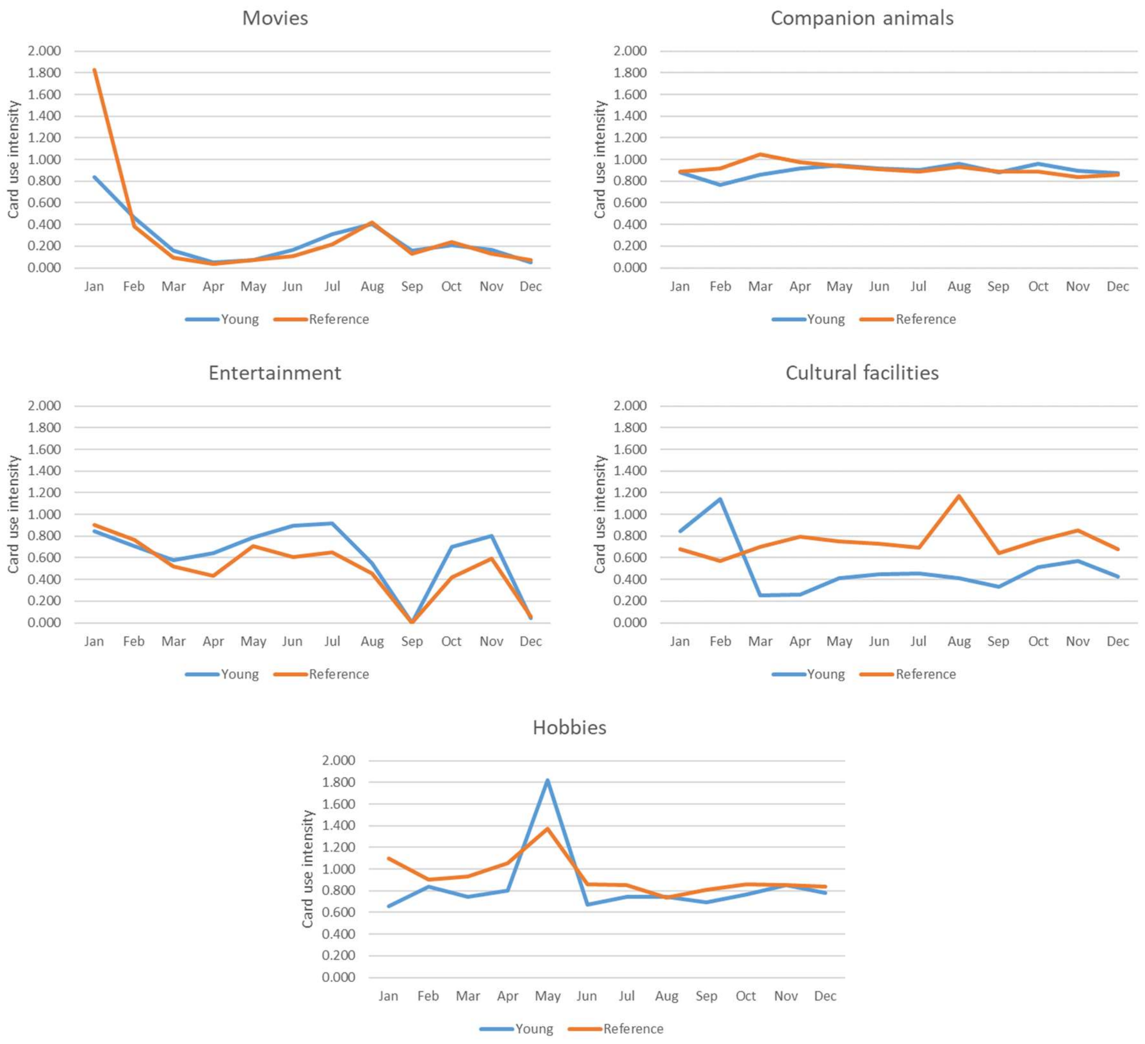

Figure F4-2. Credit card use intensity comparison by age group (sub-sectors of Recreation sector)

Figure F4-3 compares the intensity of monthly credit card use by sub-sectors in the ICT sector and by age group. Unlike in other sectors, the impact of COVID-19 is barely noticeable as there was no change in credit card usage trends, and sub-sectors such as home appliances and media even showed an increase. In particular, home appliances and mobiles experienced a temporary increase due to the Disaster Relief Fund in May. Analyzing by age group, we found that the reference group led the strong activity in sub-sectors within the ICT sector.

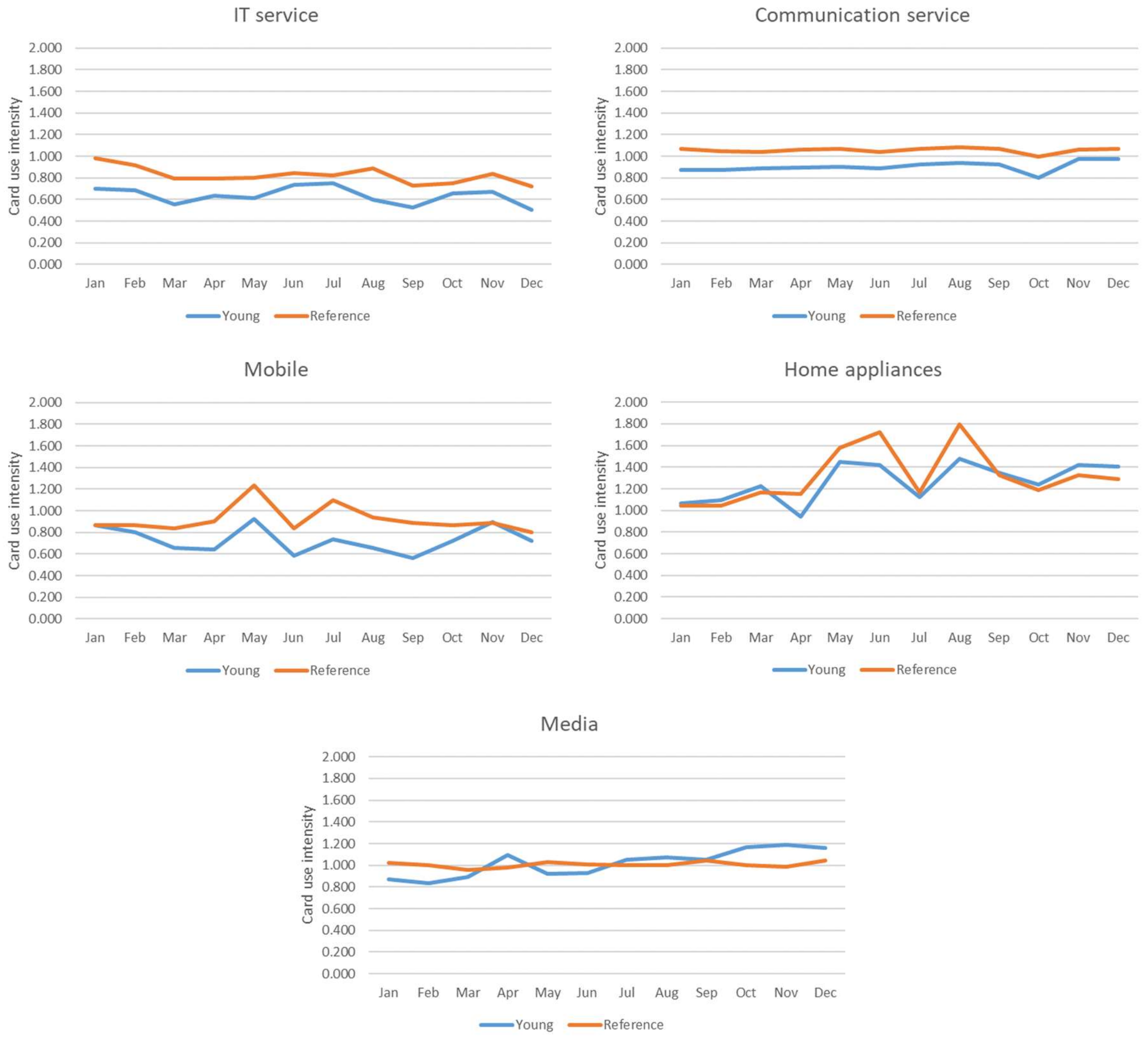

Figure F4-3. Credit card use intensity comparison by age group (sub-sectors of ICT sector)

Figure F4-4 compares the intensity of monthly credit card use by sub-sectors within the Daily Living sector and by age group. With the exception of gas stations and public transportation, overall, the level of credit card usage showed a tendency to remain steady or rise slightly, though initially there had been slight decreases in usage. This may be because the Daily Living sector has the character of industries essential for survival, while the depression experienced by gas stations and public transportation may have been caused by social distancing and working from home. In the Daily Living sector, the food and interior design sub-sectors received a temporary boost from the Disaster Relief Fund in May, and the reference group used relatively more of this fund on interior design. Analyzing by group, we found that the reference group led the sub-sectors that had an upward trend and by contrast, young people were not able to cut their spending in public transportation, a sector which showed a downtrend.

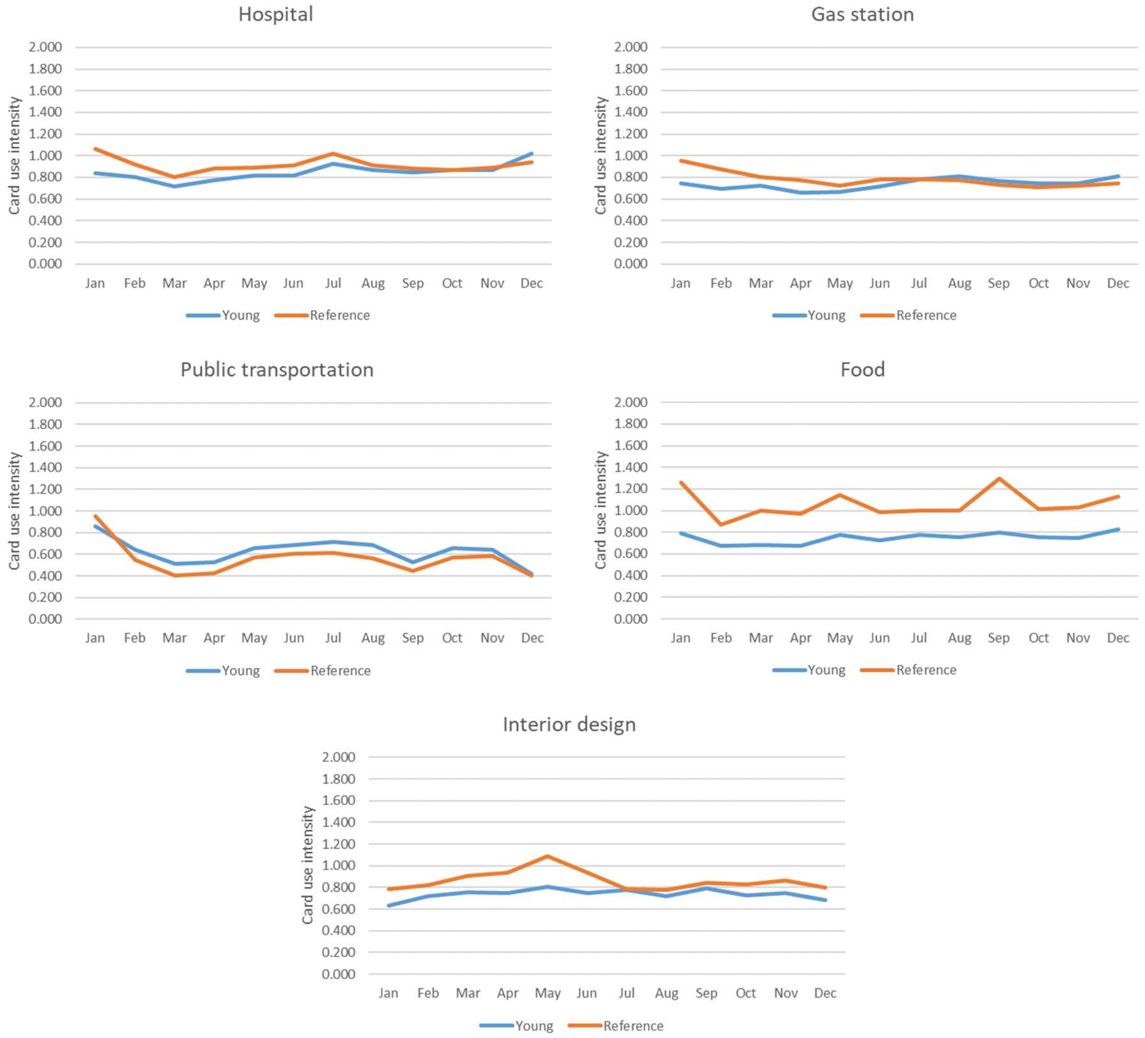

Figure F4-4. Credit card use intensity comparison by age group (sub-sectors of Daily Living sector)

Figure F4-5 compares the intensity of monthly credit card use by sub-sector and by age group in the Restaurant/Fashion sector. With the exception of the cafes sector, credit card usage declined in most sub-sectors with some variances in degree. The Disaster Relief Fund in May appears to have slightly eased the downtrend, indicating that the policy had the desired effects on this sector. With the exception of bars, the downtrend of these sub-sectors appears to have been led by young people.

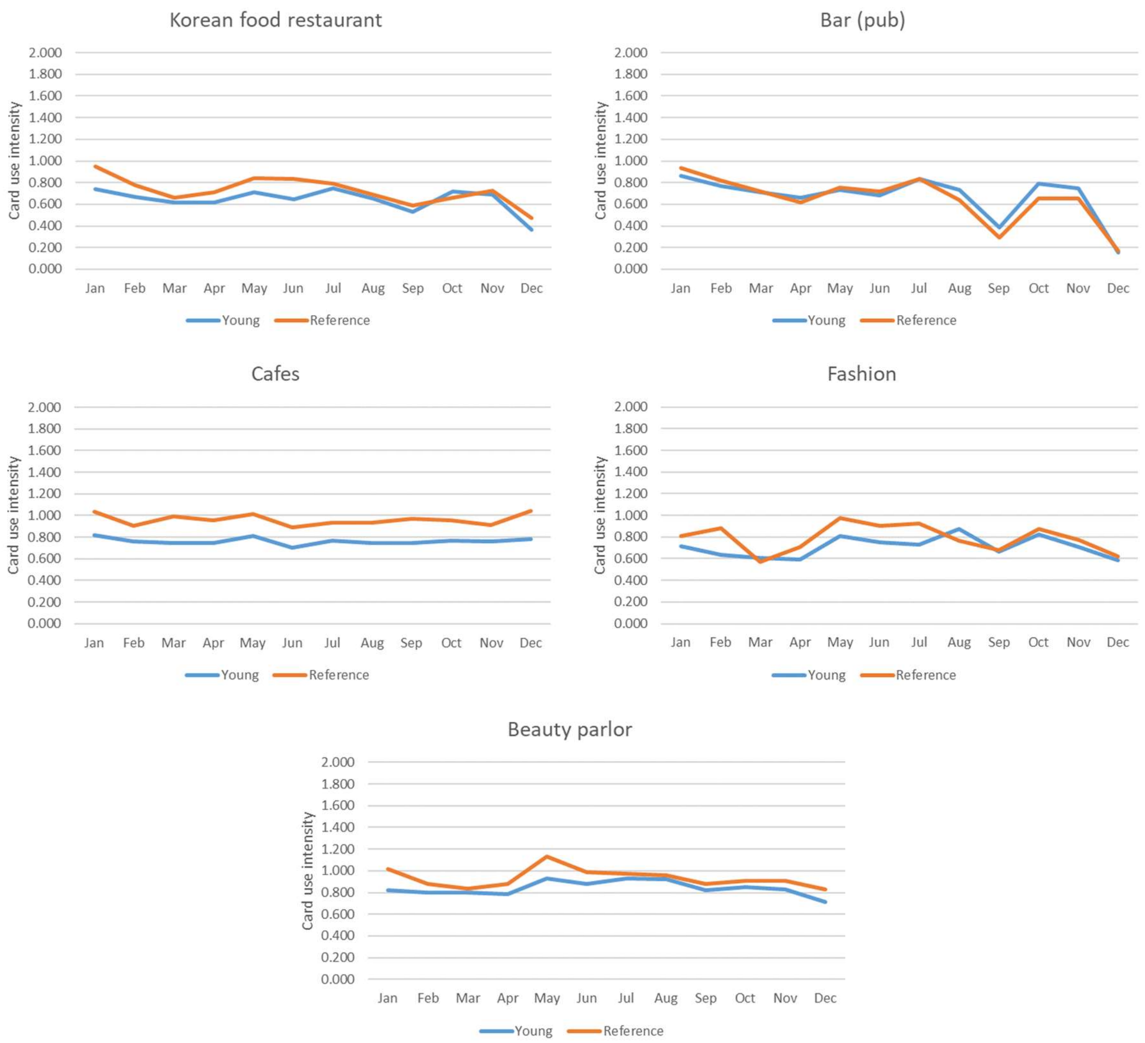

Figure F4-5. Credit card use intensity comparison by age group (sub-sectors of Restaurant/Fashion sector)

TABLE T4. COMPARISON OF MONTHLY CARD USE INTENSITY BY SUB-SECTOR AND AGE GROUP

To perform a Bayesian paired sample test, one must first define a prior distribution (probability) for hypothesis testing.[11] In this study, a normal distribution approximated to the monthly distribution of the sub-sectors in each sector was used as the prior distribution. Table T4-1 shows the descriptive statistics of the differences in paired samples for each sector and also presents the result of approximating this to 100,000 counterfactual normal distributions by simulation. We assumed the normal distribution (mean and variance) of the sample approximated by the simulation presented in Table T4-1 to be the prior distribution and proceeded to perform Bayesian inference.

Table T4-1. Descriptive statistics for the difference in paired samples for each sector and sample approximated by simulated normal distribution

Variable	N	Min.	Max.	Mean	S.D.	Skewness	Kurtosis
Leisure (20s-50s)	60	-0.69	0.30	0.010	0.191	-1.222	2.348
Leisure (20s-50s) simulation	100,000	-0.78	0.81	0.010	0.190	-0.001	-0.007
Recreation (20s-50s)	60	-0.99	0.57	-0.059	0.253	-0.980	3.276
Recreation (20s-50s) simulation	100,000	-1.09	1.02	-0.058	0.250	0.008	0.006
ICT (20s-50s)	60	-0.36	0.20	-0.113	0.135	0.388	-0.546
ICT (20s-50s) simulation	100,000	-0.73	0.45	-0.113	0.134	-0.007	-0.004
Daily Living (20s-50s)	60	0.40	1.30	0.835	0.196	-0.256	0.203
Daily Living (20s-50s) simulation	100,000	-0.69	0.60	-0.095	0.145	0.000	-0.014
Restaurant/Fashion (20s-50s)	60	-0.26	0.14	-0.087	0.098	0.266	-0.512
Restaurant/Fashion (20s-50s) simulation	100,000	-0.49	0.35	-0.088	0.097	-0.007	0.020

Table T4-2 shows the results of the Bayesian paired sample test. These results can be regarded as indicators of whether there were any significant differences after taking account of the distribution reflecting the possibility that credit card recovery may be slower in young people than in the reference group. In Table 5 and Table T4-1, it should be noted that in the Leisure sector, the recovery of young people was in fact quicker, and this trend was reflected in our results. There were 8 sub-sectors for which we determined there was substantial evidence because the Bayesian inference results were significant and the Bayesian factor was also less than 1/3.[12] According to Bayesian inference, media and bars (pubs) in addition to tourist accommodation, entertainment, and public transportation were sub-sectors with higher credit card consumption recovery among young people compared to the overall trend of the sector, while golf courses, food, and cafes were sub-sectors in which the reference group was significantly higher.

Table T4-2. Bayesian paired sample test result by sub-sector

Variable		N	Mean difference		S.D.	Bayes factor	t-statics	Degrees of freedom	Significance probability
Leisure sector	Golf courses	12	-0.206	*	0.250	0.003	-2.995	11	0.012
	Swimming pools	12	0.011		0.125	3.604	0.043	11	0.966

	Other leisure	12	0.060		0.104	2.456	1.668	11	0.123
	Tourist transportation	12	0.040		0.117	3.131	0.899	11	0.388
	Tourist accommodation	12	0.143	**	0.139	0.235	3.330	11	0.007
Recreation sector	Movies	12	-0.057		0.298	3.605	0.017	11	0.987
	Companion animals	12	-0.017		0.077	3.097	1.863	11	0.089
	Entertainment	12	0.114	**	0.134	0.270	4.437	11	0.001
	Cultural facilities	12	-0.246		0.336	0.160	-1.940	11	0.078
	Hobbies	12	-0.088		0.209	3.333	-0.496	11	0.630
ICT sector	IT service	12	-0.188	**	0.070	0.632	-3.702	11	0.003
	Communication service	12	-0.153	**	0.034	2.205	-4.122	11	0.002
	Mobile	12	-0.188		0.129	0.634	-2.017	11	0.069
	Home appliances	12	-0.050		0.153	1.083	1.419	11	0.184
	Media	12	0.013	**	0.126	0.027	3.454	11	0.005
Daily Living sector	Hospital	12	-0.068		0.073	2.985	1.270	11	0.230
	Gas station	12	-0.043		0.091	1.738	2.012	11	0.069
	Public transportation	12	0.070	***	0.059	0.003	9.750	11	<0.001
	Food	12	-0.309	***	0.094	0.000	-7.836	11	<0.001
	Interior design	12	-0.126		0.074	2.826	-1.430	11	0.180
Restaurant/ Fashion sector	Korean food	12	-0.083		0.075	3.558	0.216	11	0.833
	Bar (pub)	12	0.021	***	0.068	0.003	5.489	11	<0.001
	Cafes	12	-0.199	***	0.034	0.002	-11.369	11	<0.001
	Fashion	12	-0.081		0.101	3.522	0.212	11	0.836
	Beauty parlor	12	-0.093		0.056	3.539	-0.342	11	0.739

Note: *: $p < 0.05$, **: $p < 0.01$, ***: $p < 0.001$.

REFERENCES

1 Wilson, R. F. *et al.* Factors influencing risk for COVID-19 exposure among young adults aged 18–23 years—Winnebago County, Wisconsin, March–July 2020. *Morbidity and Mortality Weekly Report* **69**, 1497 (2020).

2 KDCA. *Coronavirus Infectious Disease-19 (COVID-19) Outbreak in Korea*, <http://ncov.mohw.go.kr/bdBoardList_Real.do?brdId=1&brdGubun=11&ncvContSeq=&contSeq=&board_id=&gubun= (in Korean)> (2021).

3 KOSIS. *Age and gender population (2019)*, <https://kosis.kr/statisticsList/statisticsListIndex.do?menuId=M_01_01&vwcd=MT_ZTITLE&parmTabId=M_01_01&parentId=A.1;A_6.2;&outLink=Y&entrType=#A_6.2 (in Korean)> (2020).

4 KOSIS. *Overall economically active population by age (2021. Jan.)*, <https://kosis.kr/statHtml/statHtml.do?orgId=101&tblId=DT_1DA7002S&conn_path=I3 (in Korean)> (2021).

5 BOK. 2019 Payment Methods and Mobile Financial Service Usage Behavior Survey Results (in Korean). (Bamk of Korea, http://ecos.bok.or.kr/, 2020).

6 CFA. *Member company management disclosure*, <https://gongsi.crefia.or.kr/portal/membership/membershipDisclosure2?companyType=1 (in Korean)> (2021).

7 BOK. *Personal card usage statistics by region and consumption type*, <http://ecos.bok.or.kr/ (in Korean)> (2021).

8 KDCA. *Plan to strengthen quarantine measures in the metropolitan area*, <http://ncov.mohw.go.kr/tcmBoardView.do?brdId=&brdGubun=&dataGubun=&ncvContSeq=359073&contSeq=359073&board_id=140&gubun=BDJ (in Korean)> (2021).

9 KDCA. *COVID-19 Regular Briefing of Central Disaster and Safety Countermeasure Headquarters* <http://ncov.mohw.go.kr/tcmBoardView.do?brdId=&brdGubun=&dataGubun=&ncvContSeq=359868&contSeq=359868&board_id=140&gubun=BDJ (in Korean)> (2021).

10 Kwon, C.-h. & Lee, H.-H. in *Proceedings of the Korean Society of Computer Information Conference.* 169-170 (Korean Society of Computer Information (in Korean)).

11 Kruschke, J. *Doing Bayesian data analysis: A tutorial with R, JAGS, and Stan*. 2nd edn, (Academic Printing & Pub., 2014).

12 Wetzels, R. *et al.* Statistical evidence in experimental psychology: An empirical comparison using 855 t tests. *Perspectives on Psychological Science* **6**, 291-298 (2011).
